# Motoneuron double discharges: only one or two different entities?

**DOI:** 10.3389/fncel.2013.00075

**Published:** 2013-05-22

**Authors:** Lydia P. Kudina, Regina E. Andreeva

**Affiliations:** Institute for Information Transmission Problems Kharkevich Institute, Russian Academy of SciencesMoscow, Russia

## Introduction

The firing behavior of spinal motoneurons is controlled by a complex interplay between the synaptic inputs that they receive and the intrinsic properties of motoneurons. In particular, the long-lasting afterhyperpolarization which follows each motoneuron spike was found to be an important mechanism in the regulation of repetitive firing behavior (Kernell, 1965, 2006). As a result, motoneurons typically fire single discharges at relatively low firing frequencies, especially in humans (about 5–20 Hz during gentle and slow voluntary muscle contractions or posture maintenance). However, under the same conditions, some motoneurons can produce a quite different type of firing termed double discharges (doublets) with uniquely short interspike intervals (ISIs), commonly ranging from 2.5 to 20 ms. Similar short ISIs were also reported under very distinct conditions, during initiation of strong and fast ballistic movements; they were referred to as doublet ISIs as well. Considering properties and underlying mechanisms of doubling, we will attempt to answer the question of whether or not this unique firing pattern can be regarded as a single entity. Note that the study of doubling in healthy humans is of interest not only in itself as a very specific motoneuron firing during natural motor control but also because it can potentially provide valuable insight into the pathophysiological mechanisms underlying doublet phenomenon that is dramatically enhanced in a number of neuromuscular diseases: proximal neuropathies (Partanen, 1978), Parkinson's disease (Baker et al., 1992), chronic cervical spinal cord injury (Thomas and Ross, 1997), spinal muscular atrophy (Rowiñska-Marciñska et al., 1999), neuromyotonia (Kleine et al., 2008), amyotrophic lateral sclerosis and Kennedy's disease (Piotrkiewicz et al., 2008; Weber et al., 2009). The importance of the doubling investigation was pointed out by the introduction of the term “double discharge” in the glossaries of terms in clinical electromyography and electrodiagnostic medicine (Simpson, 1969; AAEE, 1987; AAEM, 2001).

## Double discharges: properties, underlying mechanisms and functional significance

The firing with uniquely short ISIs was first noted in cat motoneurons by Denny-Brown (1929) and Eccles and Hoff (1932) and was termed double discharges (doublets) or doubling. Shortly thereafter, Hoff and Grant (1944) have shown that cat motoneuron doubling appeared at the start, changed to single firing as reflex drive was increased and reverted as synaptic input was diminished. The authors suggested that the doublets were characteristic of those *particular* motoneurons whose recovery cycle of the excitability after a spike included “a super-normal period” (i.e., a period of the increased excitability). Following this point, Denslow (1948) assumed that the same mechanism underlies human motor unit (MU) doublets observed during gentle voluntary contractions. Later, using intracellular recordings, Calvin and Schwindt (1972) have shown that doublets were caused by the delayed depolarization of a hump form (occurring during the falling phase of the action potential and preceding the onset of the afterhyperpolarization) that has been previously investigated in the classical studies of Granit et al. (1963) and Kernell (1964). It has been reported that under minimal firing rate the delayed depolarization hump can reach threshold and fire an additional spike, forming a doublet (Calvin and Schwindt, 1972; see also Kernell, 2006). According to Calvin (1982), the extra-spike forming a doublet is “a spike-evoked spike, i.e., two spikes for the price of one.” Thus, a doublet resulting from the hump-delayed depolarization is *post-spike* phenomenon but not a result of a large synaptic drive.

**Figure 1:**
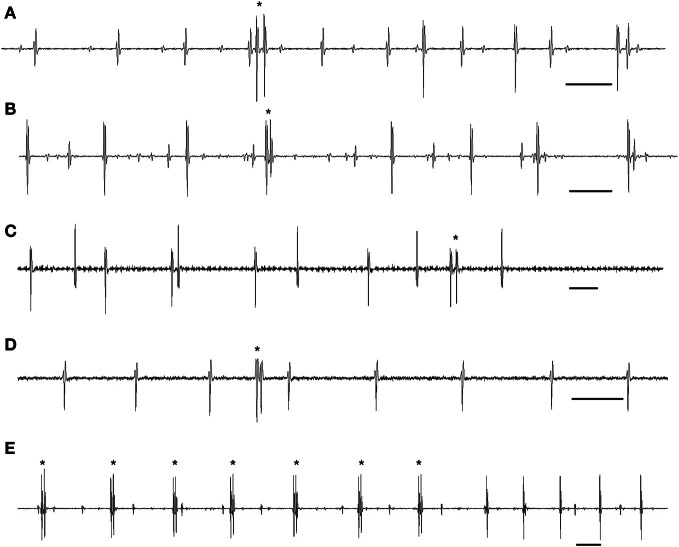
**MU single and repetitive doublets during gentle voluntary muscle contractions in humans. (A)** A doublet at motor unit recruitment. **(B)** A doublet among motor unit single-spike firing. **(C)** A doublet at motor unit de-recruitment. **(D)** A doublet of a motor unit with no background firing. **(E)** Motor unit repetitive doublet firing. **(A)** The triceps brachii. **(B–E)** The trapezius. Doublets are marked by asterisks. Time bar: 50 ms.

By analogy with cat motoneuron doublets, it has been suggested that delayed depolarization is the key mechanism underlying human MU doublet firing under conditions of a weak synaptic drive (Kudina, 1974; Bawa and Calancie, 1983; Kudina and Churikova, 1990; Garland and Griffin, 1999; Kudina and Andreeva, 2010; Duchateau and Enoka, 2011; Heckman and Enoka, 2012). In addition, it has been assumed that apart from spontaneous rises of the hump to threshold level, motoneurons are presumed to be more responsive to slight increases in excitatory synaptic input during the delayed depolarization (Kudina and Churikova, 1990; Duchateau and Enoka, 2011). Two types of doublets have been observed: single (or occasional) doublets and repetitive ones. Examples of single doublets in human MUs are presented in Figures 1A–D. As a rule, each doublet is followed by a prolonged post-doublet ISI resulting from the afterhyperpolarization summation (Calvin and Schwindt, 1972), which is important evidence for the doublet identification, indicating that the doublet was fired by a single motoneuron but not by two different motoneurons (Eccles and Hoff, 1932).

As to repetitive doublets (i.e., persistent firing with alternate short doublet and prolonged post-doublet intervals), they were briefly mentioned in both animal (Eccles and Hoff, 1932; Hoff and Grant, 1944; Calvin and Schwindt, 1972; Kirkwood and Munson, 1996; Tansey and Botterman, 1996) and human studies (Denslow, 1948; Kudina, 1974; Piotrkiewicz et al., 2008; Stephenson and Maluf, 2010). In addition, there are a few papers that have directly addressed this topic (Bawa and Calancie, 1983; Kudina and Alexeeva, 1992; Kudina and Andreeva, 2010). In these studies it was emphasized that MU repetitive doublets as well as single doublets appeared under conditions of slow, gentle voluntary contractions only. Moreover, the faster the movement, the less the probability of the appearance of repetitive double discharges (Bawa and Calancie, 1983). Instance of MU repetitive doublets is shown in Figure 1E. Concerning possible mechanisms underlying repetitive doublet firing, it has been suggested (Kudina and Andreeva, 2010) that the mechanism is complex and likely includes a hump-delayed depolarization as the primary determinant that becomes persistent due to the activation of a plateau potential that can markedly alter motoneuron excitability (for review see Heckman et al., 2009).

As noted above, the term “doublets” was introduced in the first doublet reports under conditions of a weak synaptic input. Such doublets were termed “true” doublets in the seminal report of Bawa and Calancie (1983). However, in the current literature, short ISIs that were recorded in human MUs at the beginning of strong or very fast (ballistic) movements (e.g., Desmedt and Godaux, 1977; Van Cutsem et al., 1998; Christie and Kamen, 2006) or in rat motoneurons in response to strong step of injected current (Mrówczyński et al., 2010) were also referred to as doublet ISIs, in spite of sharp contrast in conditions of their appearance and distinct (often opposite) properties. In contrast to true doublets, the short ISIs above were recorded under *large* excitatory drive and therefore they probably correspond to motoneuron firing in the “secondary range” (Granit et al., 1966), i.e., these short ISIs are *post-synaptic* phenomena. Thus, there is little evidence to support the suggestion about a common mechanism (in particular, the delayed depolarization) underlying *all* short ISIs (e.g., Christie and Kamen, 2006). The opinion is consistent with the previous conclusion of Bawa and Calancie (1983) that given two types of short ISIs arise from two distinct spinal phenomena. The authors have purposely refrained from using the term doublet in context of ballistic movements. In order to distinguish these short ISIs from those of true doublets, the former will be further called “like-doublet ISIs.”

As to ISI durations of both kinds of doublets, although their ranges commonly coincide but the quite different character of the firing is clearly revealed by plotting ISI histograms that exhibit a unimodal distribution for firing with initial short ISIs of like-doublets and a bimodal distribution for firing with true doublets forming a separate ISI group that was well outside the ISIs of single-spike firing (Kudina, 1974; Kudina and Andreeva, 2010).

Further, true doublets were only found in some MUs and each doublet (single or repetitive) was typically followed by a prolonged post-doublet ISI. Subjects can improve in their ability to recruit MUs with repetitive doublets but they were never able to change the ISI durations of true doublets, since the doublet ISI appeared to be completely independence of the will of the subject (Bawa and Calancie, 1983; Kudina and Andreeva, 2010). In contrast, like-doublets were recorded in most (if not all) motoneurons under study (e.g., Mrówczyński et al., 2010). As described by Van Cutsem et al. (1998), under ballistic contractions, one to three short ISIs can arise in succession without prolonged post-doublet ISIs and ISI durations could be decreased by training. It should be point out that firing with true doublets could display repetitive doublet series while like-doublet firing never showed ones.

With regard to the functional significance of single and repetitive true doublet firing, currently it can only be speculated upon. In the late 1990s, Garland and Griffin (1999) noted that “At present it is impossible to tell whether double discharges represent a functional entity, an enterprise involving some form of motor control strategy. Conversely, double discharges may be nothing more than a statistical anomaly, in that all MNs may discharge with doublets occasionally.” Presently, the functional significance of double discharges remains uncertain as well (Stephenson and Maluf, 2010; Duchateau and Enoka, 2011; Heckman and Enoka, 2012). At first sight, it would seem that any short ISI must greatly enhance force production of a muscle. The suggestion is based on the evidence that short ISIs have been observed at the onset of strong and ballistic contractions (e.g., Desmedt and Godaux, 1977; Van Cutsem et al., 1998; see also Garland and Griffin, 1999). However, this role may be suggested as a pivotal role for like-doublets only. As to true doublets, it does not seem likely that during slow gentle voluntary contractions, a *single* doublet of *a single* MU can result in increasing muscle force, whereas the other MUs fire single discharges at low firing rates. Furthermore, a prolonged post-doublet ISI must invalidate the effect of a short interval, even if it is present. Yet more unlikely is the suggestion about the extra-force generation by a doublet occurring just before an MU de-recruitment when the subject stops the muscle contraction. We suggest, therefore, that single doublets occasionally occurring in *some* MNs are hardly functionally important, rather, in fact, they “may be nothing more than a statistical anomaly” (Garland and Griffin, 1999) or, in the other words, nothing more than “a price for the organization” (for possessing the hump-delayed depolarization). In contrast, persistent repetitive doublet firing (particularly characteristic of postural muscles) appears likely to be a result of a certain form of motor control during postural tasks. For example, it could be suitable for motor tasks which, after MU starting, involves a strategy of “self-regulation,” i.e., no active control by the subject is needed for their support.

## Concluding remarks

We conclude that motoneuron firing including similar short ISIs (doublets) can possess quite different essential properties and underlying mechanisms. Therefore, there is little evidence to support the point considering only one entity for doublet firing pattern. In our opinion, true doublets and like-doublets represent, in the fact, rather different kind of motoneuron firing behavior. If so, using one and the same term “doublets” for description of both inevitably leads to misleading interpretations. Based on our data and the point of Bawa and Calancie (1983), we suggest not using the term “doublet” for short ISIs resulting from strong excitatory drive. This will allow most of the seemingly disparate results in the literature to be reconciled.
